# COX-2 in cancer: Gordian knot or Achilles heel?

**DOI:** 10.3389/fphar.2013.00034

**Published:** 2013-03-28

**Authors:** Ioannis Stasinopoulos, Tariq Shah, Marie-France Penet, Balaji Krishnamachary, Zaver M. Bhujwalla

**Affiliations:** Johns Hopkins University In-vivo Cellular and Molecular Imaging Center Program, Division of Cancer Imaging Research, The Russell H. Morgan Department of Radiology and Radiological Science, The Johns Hopkins University School of MedicineBaltimore, MD, USA

**Keywords:** cyclooxygenase 2, inflammation and cancer, inflammation and hypoxia, COX-2 and HIF-1, COX-2 and LEF-1, invasion, metastasis, COX-2 inhibitors

## Abstract

The networks of blood and lymphatic vessels and of the extracellular matrix and their cellular and structural components, that are collectively termed the tumor microenvironment, are frequently co-opted and shaped by cancer cells to survive, invade, and form distant metastasis. With an enviable capacity to adapt to continually changing environments, cancer represents the epitome of functional chaos, a stark contrast to the hierarchical and organized differentiation processes that dictate the development and life of biological organisms. The consequences of changing landscapes such as hypoxia and acidic extracellular pH in and around tumors create a cascade of changes in multiple pathways and networks that become apparent only several years later as recurrence and metastasis. These molecular and phenotypic changes, several of which are mediated by COX-2, approach the complexities of a “Gordian Knot.” We review evidence from our studies and from literature suggesting that cyclooxygenase-2 (COX-2) biology presents a nodal point in cancer biology and an “Achilles heel” of COX-2-dependent tumors.

## INFLAMMATION IN THE TUMOR MICROENVIRONMENT

**FIGURE 1:**
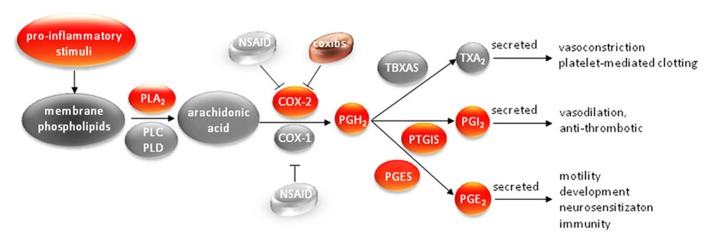
**The cyclooxygenase pathway.** In response to pro-inflammatory stimuli COX-2 is expressed in the cytoplasm of many cell types where it catalyzes the conversion of arachidonic acid to prostaglandins and thromboxanes. PL, phospholipase; NSAID, non-steroidal anti-inflammatory drugs; coxibs, COX-2-selective inhibitors; COX, cyclooxygenase; PG, prostaglandin; TX, thromboxane; PTGIS, prostacyclin synthase; PGES, prostaglandin synthase.

Hostile physiological environments such as hypoxia and acidic extracellular pH which exist in solid tumors, as well as environments created by conventional therapy such as radiation, chemotherapy, and surgery, may promote invasion and metastasis through inflammatory responses and the formation of eicosanoids. As outlined in the schematic in **Figure 1**, the characteristic response of living vascularized tissue to injury is inflammation, which induces the formation of eicosanoids. Three classes of phospholipases (PLs) A_2_, C, and D, participate in the formation of free arachidonic acid (AA) from membrane phospholipids in response to mechanical, chemical, and physical stimuli ( Kaiser et al., 1990). Since AA is derived from membrane phospholipids, its production and utilization in the formation of eicosanoids is closely coupled to membrane choline phospholipid metabolism ( Kaiser et al., 1990). In response to pro-inflammatory cytokines AA is converted to various eicosanoids by the action of cytochrome P450 enzymes, lipoxygenases, and cyclooxygenases (COX;  Needleman et al., 1986;  Haeggstrom et al., 2010;  Greene et al., 2011). These eicosanoids impact cell motility, invasion, vascular characteristics, and metastatic dissemination ( Fulton, 1988;  Liu et al., 2010;  Menter et al., 2010). Most solid tumors, including breast cancers, exhibit inflammatory properties characterized by increased levels of prostaglandins (PGs) and other pro-inflammatory molecules that are secreted by tumor cells, stromal cells, and specialized immune cells during inflammation, with nuclear factor kappaB (NF-κB) considered as a central molecular mediator of these responses ( DiDonato et al., 2012). Such an upregulation of inflammatory characteristics is not surprising in view of the similarities between physiological conditions in injured tissue, such as hypoxia and low extracellular pH, and the physiological environment of solid tumors ( Gillies et al., 2000). Unlike lung and colon, where the source of inflammatory signaling as an instigator of, and contributor to, tumorigenesis is obvious, the breast has long been thought of as lacking such extrinsic inflammatory stimuli. Epidemiological studies however, have long showed a link between obesity and breast cancer ( Gilbert and Slingerland, 2013). Adipose tissue, abundant in the human breast, has been shown to secrete pro-inflammatory cytokines, termed adipokines, capable of producing low-grade chronic inflammation in the human breast ( Ouchi et al., 2011;  Baumgarten and Frasor, 2012).

## COX-2 IN BASIC, TRANSLATIONAL, AND CLINICAL BREAST CANCER RESEARCH

COX-1 and COX-2 are cytoplasmic enzymes that convert PLA_2_-mobilized AA into the lipid signal transduction molecules PGs and thromboxanes (TXs;  van der Donk et al., 2002). One major product of the COX-2-catalyzed reaction is PGE_2_, an inflammatory mediator participating in several biological processes, including development, pain, immunity, angiogenesis ( Smith et al., 2000), and cancer ( Howe, 2007;  Singh-Ranger et al., 2008). Studies examining the expression of COX-2 using immunohistochemistry concluded that COX-2 expression is observed in approximately 42% of breast cancers ( Glover et al., 2011). COX-2 function has been the target of pharmaceutical intervention in a multitude of widespread degenerating conditions, including autoimmune diseases, gastric inflammation, and several different cancers, such as colon, gastric, breast, and lung cancer ( Koehne and Dubois, 2004;  Xu et al., 2004;  Wallace, 2005;  Wang et al., 2005;  Diaz-Cruz and Brueggemeier, 2006;  Krysan et al., 2006). Its expression is induced by pro-inflammatory cytokines, such as interleukin (IL)-1β and tumor necrosis factor (TNF)-α, and its promoter contains a cyclic AMP response element, a NF-κB binding site, and a nuclear factor for interleukin-6/CCAAT enhancer-binding protein (NF-IL6/C/EBP) sequence ( Chun and Surh, 2004).

The utility of COX-2 as a target for cancer treatment has been debated for decades and the first clinical trials using COX-2-selective inhibitors for cancer treatment took place in the late 1990s when celecoxib was shown to reduce colon adenomas in patients with familial adenomatous polyposis (FAP;  Steinbach et al., 2000). Soon thereafter, celecoxib was shown to reduce polyp formation in sporadic colorectal adenocarcinomas ( Arber et al., 2006;  Bertagnolli et al., 2006), but with increased risk of death by cardiovascular complications ( Baron et al., 2008;  Solomon et al., 2008). As a result, celecoxib use for cancer prevention was limited to FAP patients. Results from clinical trials using celecoxib alone suggested a modest effect of celecoxib in primary breast cancer ( Martin et al., 2010). Studies using celecoxib in combination with aromatase inhibitors were either terminated early due to cardiovascular side effects ( Falandry et al., 2009) or showed no significant difference in the response rate with the inclusion of celecoxib ( Chow et al., 2008). The cardiovascular side effects observed following prolonged celecoxib administration were attributed to an imbalance of eicosanoid production toward the pro-thrombotic TXA_2_ ( Antman et al., 2007). The limited responses to celecoxib coupled with the significant cardiovascular side effects have resulted in a significant shift in focus to downstream targets such as PG synthases and receptors. Initially, it was reasoned that celecoxib has many COX-2-independent functions that are responsible for its anti-tumorigenic effects ( Grosch et al., 2006) and that, given the cardiotoxicity associated with COX-2 inhibition, the COX-2-independent anti-tumorigenic effects of coxibs needed to be pursued further. Attempts to reduce inflammation using anti-inflammatory celecoxib analogs designed to not bind to COX-2 and display anti-tumor and anti-inflammatory properties have shown some promise ( Schonthal et al., 2008).

Despite the discordance between the promise of basic studies and the limited clinical benefits of coxibs, several observations favor COX-2 as a target for cancer treatment. First, targeting pathways downstream of COX-2 is likely to dilute the effect of COX-2 inhibition, since the COX reaction is the rate-limiting enzyme of prostanoid formation ( Samuelsson et al., 1978). Second, there is compelling evidence obtained by studying the effects of COX-2 using short interfering RNA (siRNA) or using the exogenous supplementation of COX-2 reaction products demonstrating that COX-2 promotes carcinogenesis and metastasis. Such evidence is discussed below and in the references cited. Third, TXA_2_ synthase inhibitors given concurrently with COX-2 inhibitors could alleviate the cardiovascular side effects attributed to the inhibition of COX-2 by coxibs. A more detailed review of the risks and rewards of targeting COX-2 in cancer was recently published ( Menter et al., 2010). Fourth, the limited benefits of celecoxib in human subjects can be explained by the observation that many of the coxib-associated effects observed *in vitro* and *in vivo* are not related to COX-2 inhibition, but to COX-2-independent actions of coxibs ( Grosch et al., 2006;  Schonthal et al., 2008). Fifth, not all tumors or metastatic processes are COX-2-dependent and the expression of a highly inducible enzyme such as COX-2 does not necessarily suggest critical function in every instance it is observed. Thus the utilization of COX-2 inhibitors, even if they specifically inhibited COX-2 function, would not be beneficial until primary tumors and metastatic processes that had a significant requirement for COX-2 were targeted. It would thus be of clinical benefit to discover biomarkers that reflect the activity of COX-2 in tumors and in the tumor microenvironment.

## COX-2 EXPRESSION AND CLINICAL OUTCOMES IN BREAST CANCER

Several studies have sought to correlate the expression of COX-2 with existing clinical markers in breast cancer. Recently, a large study (*n* = 1162) of biomarker expression in ductal carcinoma *in situ* (DCIS) was published ( Kerlikowske et al., 2010) where it was shown that the diagnosis of breast tumors by palpitation or the concurrent triple expression of p16/COX-2/Ki67 signified an increased risk of recurrence of invasive breast cancer 8 years following initial diagnosis and lumpectomy. A separate study of 248 cases of breast cancer showed that COX-2 expression was elevated in hormone receptor (HR) negative or human epidermal growth factor receptor 2 (HER2) positive subpopulations and correlated with an activation of the oncogene Akt and with poor survival ( Glover et al., 2011). Others, however, demonstrated that COX-2 expression correlates with poor outcomes independently of the expression of established markers of breast cancer ( Kim et al., 2012). In addition, COX-2 expression has been demonstrated across all clinically useful categories of breast cancers suggesting that COX-2 expression is not predominantly related to hormone or HER2 receptor status. Further complicating the retrofitting of COX-2 positivity within established breast cancer subtypes is the fact that COX-2 expression and function may originate from non-epithelial cellular components of the microenvironment such as the immune response, or the tissue response to injury. Correlative studies that attempt to stratify the expression of COX-2 within current types of breast cancer would miss the transient influence of microenvironment-derived COX-2. It is our view that the discovery of biomarkers that predict the mechanistic association of breast tumor initiation, progression, and metastasis with COX-2 function, can only be attained by the employment of high-throughput/omics approaches on a variety of constituent and representative cells that are engineered to over- or under-express COX-2. The objective would be to derive tumor-promoting COX-2-associated molecular signatures that can be correlated with aggressive phenotypes in experimental animal models and validated in sample tissue or sera of patients.

## COX-2 INDUCES THE EXPRESSION OF ONCOGENES BY CO-OPTING BIOLOGICAL EFFECTORS OF HYPOXIA AND DEVELOPMENT

**FIGURE 2:**
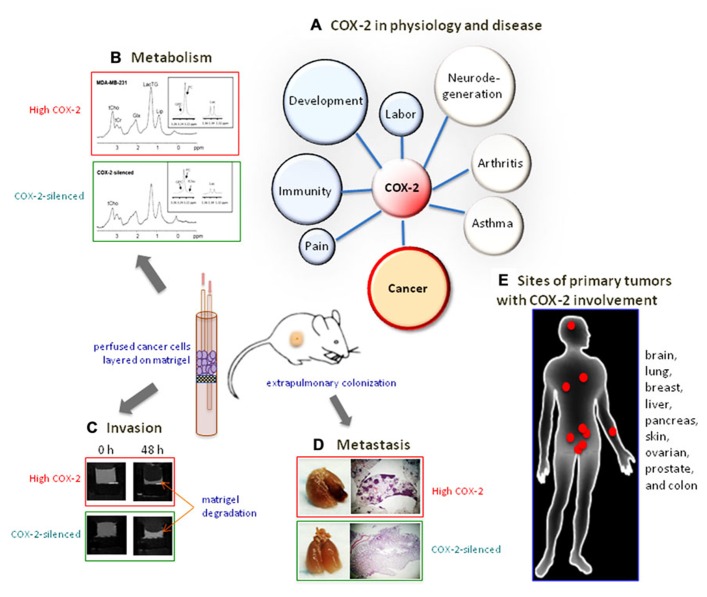
**COX-2, Gordian knot or Achilles heel?** **(A)** Physiological processes with significant COX-2 involvement. **(B)** COX-2 silencing changes the metabolic profile of breast cancer cells to a less aggressive phenotype. **(C)** COX-2 silencing reduces the invasiveness of breast cancer cell. **(D)** COX-2 silencing abolishes the extrapulmonary colonization of metastatic breast cancer cells. **(E)** Sites of primary tumors with extensive COX-2 involvement.

Given the pleiotropic effects of COX-2 products during development, physiology and disease we have sought to investigate whether COX-2 represents a Gordian knot or an Achilles heel in breast cancer by utilizing COX-2-specific siRNA in a cell-based model of tumor growth and metastasis (summarized in **Figure 2**). We have observed increased expression of COX-2, in several, but not all, triple negative human breast cancer cells that were also metastatic (unpublished observations). We silenced COX-2 in the most metastatic breast cancer cells and observed a profound decrease of metastasis and tumor onset *in vivo*, although cell proliferation rates were unaffected in culture ( Stasinopoulos et al., 2007). Interactions between the cancer cell and the tumor microenvironment (TME) following COX-2 silencing became apparent in functional imaging assays that revealed a significant decrease of invasion into reconstituted extracellular matrix (ECM;  Stasinopoulos et al., 2007;  Shah et al., 2012), an altered interaction between endothelial cells and cancer cells following COX-2 silencing ( Stasinopoulos et al., 2008), and a significant alteration in glycolysis, pH, and choline metabolism ( Stasinopoulos et al., 2008;  Shah et al., 2012). The associations between COX-2 and choline metabolism, glycolysis and pH have identified new functional roles of COX-2 that may reveal new biomarkers and new targets to use in combination with COX-2 targeting.

The activation of several genes that form the adaptive response of cells to hypoxia is mediated through the binding of hypoxia-inducible factor (HIF)-1 to the hypoxia response elements that regulate the transcription of these genes ( Maxwell et al., 1997). Under oxygenated conditions HIF-1α is rapidly degraded, but under hypoxic conditions HIF-1α is stabilized ( Liu and Semenza, 2007). We examined differences in the hypoxia and inflammation-driven functional activation of HIF-1α in COX-2-expressing and COX-2-silenced cells, and found that COX-2 is important for IL-1β, but not hypoxia-driven, HIF-1α stabilization and induction of HIF-1α target genes ( Stasinopoulos et al., 2009). These data imply that PGE_2_ can employ the transcription factor HIF-1, and the multitude of HIF-1 responsive genes, to promote malignant phenotypes associated with HIF-1 activation such as drug resistance, increased invasion, and altered metabolism ( Semenza, 2012;  Shay and Simon, 2012) even under well-oxygenated conditions. Choline kinase (Chk), a HIF-1-regulated ( Glunde et al., 2008) cytoplasmic enzyme responsible for the phosphorylation of choline to phosphocholine (PC) involved in invasion and metastasis ( Glunde et al., 2011), was also down-regulated in COX-2-silenced cells ( Shah et al., 2012), suggesting a possible mechanism of regulation of phospholipid metabolism by the COX-2-HIF-1 axis. Our results are compatible with a study showing that IL-1-mediated HIF-1 stabilization *via* COX-2 upregulation and NF-κB activation in lung and colon cancer cells ( Jung et al., 2003). Cancers with a strong inflammatory component will most likely have functional HIF-1α activation even under normoxic conditions; targeting COX-2 could minimize these effects. Several insults to tissue such as reactive oxygen species, ionizing radiation, and physical trauma during surgery are known stimuli for the initiation or exacerbation of the inflammatory response ( Molla and Panes, 2007;  Rundhaug and Fischer, 2008). Peri-operative administration of the COX-2 inhibitor etodolac is being investigated in clinical trial NCT00502684. Our data support the administration of anti-inflammatory agents immediately following surgery and ionizing radiation treatment of patients to minimize activation of the IL-1β–COX-2–HIF-1α axis of oncogenic signaling. This topic has been extensively discussed elsewhere ( Choy and Milas, 2003;  Imtiyaz and Simon, 2010). Transcriptome analysis revealed differential expression of genes that control angiogenesis, invasion, and differentiation including the wnt/β-catenin pathway ( Stasinopoulos et al., 2012). These changes will identify candidate reporter elements in the promoter of these genes that can be used to image the induction of COX-2 expression. Loss of COX-2 resulted in the loss of lymphoid enhancer-binding factor-1 (LEF-1) mRNA, and nuclear LEF-1 protein, while exogenous supplementation of PGE_2_ restored nuclear LEF-1 levels in COX-2-silenced cells ( Stasinopoulos et al., 2012). Since LEF-1 is a transcription factor that mediates Wnt signaling during development and disease, these results are consistent with the demonstration that PGE_2_ can promote non-canonical Wnt signaling by directing the translocation of β-catenin from the cytoplasm to the nucleus of colon cancer cells ( Castellone et al., 2005). The induction of LEF-1 and the stabilization of HIF-1α by COX-2 provide additional examples of the co-option of molecular pathways central to the response to injury and development by tumors. In addition to providing important cues regarding the role of COX-2 in breast cancer, transcriptome analysis of COX-2-silenced and COX-2 containing cells has indicated candidate reporter elements in the promoters of these genes that can be used to image the function of COX-2 *in vivo*.

## SILENCING OF COX-2 INHIBITS METASTASIS AND DELAYS TUMOR ONSET OF POORLY DIFFERENTIATED METASTATIC BREAST CANCER CELLS

Breast cancer cells silenced for the expression of COX-2 using stable expression of short hairpin RNA were less able to invade reconstituted ECM than parental cells *in vitro* ( Stasinopoulos et al., 2007). MDA-MB-231 cells silenced for COX-2 expression showed reduced mRNA expression of several oncogenic markers, including IL-11, a marker for metastasis of breast cancer to bone, the Notch1 receptor ligand JAG1, whose expression is correlated with poor breast cancer prognosis, CXCR4, a receptor involved in cancer cell invasion, and matrix metalloproteinase-1 (MMP-1), a secreted enzyme responsible for the degradation of the stroma during breast cancer cell invasion. Dynamic tracking of invasion and metabolism of COX-2-silenced intact MDA-MB-231 cells, using our magnetic resonance (MR) compatible cell perfusion apparatus, under controlled pH, temperature, and oxygenation over 48 h, revealed significantly reduced levels of total choline, PC, and lactate compared to parental MDA-MB-231 cells (**Figure 2B**) and reduced invasion (**Figure 2C**). These changes also correlated with a reduction of Chk levels in COX-2-silenced cells ( Shah et al., 2012). The metabolic changes are consistent with a less aggressive phenotype since PC and total choline, as well as Chk, are biomarkers of malignancy ( Glunde et al., 2011).

Loss of COX-2 resulted in the significant delay of tumor onset when the cells were injected in the mammary fat pad of severe combined immunodeficient (SCID) mice ( Stasinopoulos et al., 2007) consistent with the observation that COX-2 was found to be a part of a gene signature that predicted metastasis of MDA-MB-231 cells to the lung ( Gupta et al., 2007). Silencing of COX-2 resulted in the inhibition of metastasis to the lungs of SCID mice after intravenous injection ( Stasinopoulos et al., 2007) and **Figure 2D**. Our results show that COX-2 expression modulates the expression or function of many ECM components, including collagen, glycoproteins [e.g., thrombospondin-1 (THBS-1)], hyaluronan, and proteoglycans (e.g., lumican). It is possible that tumor-derived COX-2 modifies the ECM enabling tumors to successfully establish metastases. The role of COX-2 and COX-2-produced PGs in promoting cancer cell adhesion in the ECM has recently been reviewed elsewhere ( Menter and Dubois, 2012). Additionally, evidence that COX-2 inhibition reduces collagen deposition, tumor growth, and invasion during mammary gland involution was recently described ( Lyons et al., 2011). While this model specifically addressed the increasing risk of breast cancer following pregnancy, the association of collagen deposition and remodeling with breast cancer metastasis is under investigation ( Schedin and Keely, 2011). It was recently shown that increased collagen content correlated with metastasis to lymph nodes ( Kakkad et al., 2012). Alignment of collagen fibers perpendicularly to the tumor boundary was associated with decreased disease-free survival in breast cancer patients ( Conklin et al., 2011). Further studies are needed to understand the role of COX-2 in the ECM remodeling during normal development and disease. Modification of the ECM by microenvironment-derived COX-2 could also explain the observation that tissues with active inflammatory processes, such as wounds, are sites of frequently successful metastases. These data strongly support investigating the relationship between COX-2 and the structure and function of the ECM further.

## THE MALIGNANT PHENOTYPE OF BREAST CANCER CELLS IS REDUCED BY COX-2 SILENCING

COX-2 silencing resulted in the loss of expression of metabolic symporters, ECM components, and several proangiogenic factors ( Stasinopoulos et al., 2008). Our data implicate COX-2 as a regulator of processes central to tumor metabolism, angiogenesis, and ECM composition, making it a major contributor to a microenvironment permissive to tumorigenesis, invasion, and metastasis. In these functional studies, silencing COX-2 expression resulted in a decrease of lactate production or export, a decrease in medium acidification, a decrease in the secretion of the ECM component hyaluronan, and an inhibition of human umbilical vein endothelial cell (HUVEC) network formation ( Stasinopoulos et al., 2008). Extracellular lactate concentration and extracellular acidification were reduced in COX-2-silenced cells. Microarray results from COX-2-expressing and COX-2-silenced cells revealed alterations in transcripts regulating glutamate transport (SLC1A1) as well as other glycolysis-related transporters and enzymes involved in cancer progression such as hexokinase II. COX-2-silenced MDA-MB-231 cells actively inhibited HUVEC network formation when co-cultured on an ECM gel suggesting that COX-2 plays an important role in angiogenesis, a process essential for primary and metastatic tumor growth ( Potente et al., 2011). Transcriptome comparisons between COX-2-expressing and COX-2-silenced cells revealed changes in several angiogenesis related transcripts such as CXCR4 ( Stasinopoulos et al., 2007) confirmed at the protein level ( Stasinopoulos et al., 2008). CXCR4 is a chemokine receptor important in cancer cell invasion and angiogenesis and has been shown to be regulated by COX-2 and PGE_2_ levels in Lewis lung carcinoma cells ( Katoh et al., 2010). The loss of several proangiogenic factors may explain the inability of HUVEC to form networks and self-associate and the marked reduction in orthotopic tumor growth. COX-2 null mice that overexpressed a HER2/neu transgene under the control of the mouse mammary tumor virus promoter, showed reduced tumor multiplicity and size compared to COX-2 expressing mice, but also demonstrated reduced vasculature in non-tumor mammary tissue ( Howe, 2007). Conversely, forced COX-2 expression in the mammary gland of mice increased microvascular density, which was reversed by treatment with celecoxib ( Chang et al., 2004). Many COX-2 reaction products have been implicated in angiogenesis including TXA_2_, PGI_2_, and PGE_2_ ( Pradono et al., 2002;  Chang et al., 2004;  Obermajer et al., 2011). Imaging the COX reaction directly, or indirectly through the effects of the COX-2 products *in vivo*, is ideally compatible with identifying mechanisms employed by COX-2-dependent tumors, and identifying markers of COX-2-promoted angiogenesis and metastasis.

## CONCLUSION

Molecular characterization and functional imaging have identified new functional roles for COX-2, creating new possibilities for more effective COX-2 targeting, and for imaging COX-2 expression and activity. Our results are presented within the context of the function of COX-2-related biology and disease in **Figure 1**. Here we have highlighted the importance of targeting this pathway in cancer, and establishing strategies to image COX-2 expression and activity.

These studies emphasize the importance of expanding our understanding of the role of COX-2 in altering the tumor phenotype and of non-invasively identifying tumors that have increased COX-2 expression and functionality, to select for COX-2 targeting. The answer to the question posed in this title cannot be decided until COX-2-dependent tumor initiation, growth, or metastasis is identified and inhibited *in vivo* using novel and improved approaches.

## Conflict of Interest Statement

The authors declare that the research was conducted in the absence of any commercial or financial relationships that could be construed as a potential conflict of interest.
